# Clinical Efficacy and Mechanisms of ZiyuShuang, an Anti‐Skin Aging Formula Identified Through Network Medicine Framework Analysis

**DOI:** 10.1111/jocd.71171

**Published:** 2026-09-23

**Authors:** Yingpeng Tong, Xiangmei Ni, Xinnan Jiang, Ru Zhang, Pengcheng Guo, Chunbo Feng, Jianxin Wang

**Affiliations:** ^1^ School of Advanced Study, School of Pharmaceutical Sciences Taizhou University Taizhou China; ^2^ Zhejiang Provincial Key Laboratory of Evolutionary Ecology and Conservation Taizhou University Taizhou China; ^3^ R&D Center Shanghai Jahwa United Co. Ltd. China; ^4^ Department of Pharmaceutics, School of Pharmacy Fudan University Shanghai China; ^5^ Key Laboratory of Smart Drug Delivery Ministry of Education Shanghai China; ^6^ Quzhou Fudan Institute Quzhou Zhejiang Province China

**Keywords:** clinical trial, network medicine analysis, network pharmacology, skin aging, ZiYuShuang

## Abstract

**Background:**

Skin aging, a key aspect of physiological decline, markedly affects appearance, making strategies to delay it vital for quality of life.

**Materials and Methods:**

We applied network medicine analysis to screen herbal medicines from the TCMSP database, yielding an anti‐skin aging formula, ZiYuShuang (ZYS). Its efficacy and mechanisms were evaluated using network pharmacology, in vitro assays, and clinical trials.

**Results:**

Of 155 herbal medicines identified with anti‐skin aging potential from TCMSP, Qi‐reinforcing drugs were most effective. Informed by literature and preliminary data, we developed ZYS. Network pharmacology linked ZYS's effects to Focal Adhesion, Relaxin Signaling, PI3K‐Akt, TNF, and Apoptosis pathways. In vitro, ZYS regulated elastin, collagen (types I, III, IV, VII), and decorin expression. Clinically, ZYS enhanced skin hydration, glossiness, transparency, dermal density, and thickness, while reducing redness, yellowness, firmness, roughness, and wrinkle severity (forehead lines, nasolabial folds, crow's feet, and total facial lines).

**Conclusion:**

ZYS, derived from network medicine, emerges as a potent adjunct for skin aging interventions.

## 1 Introduction

Skin aging is a complex, irreversible biological process driven by intrinsic and extrinsic factors [1]. Intrinsic aging stems from chronological progression and physiological changes [2], while extrinsic aging results from ultraviolet (UV) radiation [3], smoking [4], environmental exposures [5], and harmful chemicals [6]. Extrinsic triggers, in particular, cause molecular damage to skin cells, impairing function and leading to rough texture, wrinkles, reduced elasticity, pigmentation, epidermal sagging, and thinning of the dermis and epidermis [7].

Collagen, a key structural protein, underpins skin integrity and elasticity. Multiple collagen types—including COL1A1, COL1A2, COL3A1, COL4A1–COL4A5, and COL7A1—form the extracellular matrix (ECM) and are implicated in skin aging [8, 9, 10, 11, 12]. Integrin alpha 1 (ITGA1) and fibronectin (FN1) mediate cell adhesion and ECM interactions, with altered expression affecting cell migration, proliferation, and differentiation during aging [13, 14]. Matrix metalloproteinases (MMPs), such as MMP1, degrade collagen and ECM components, with activity rising with age and UV exposure, accelerating skin aging [15]. Filaggrin (FLG), critical for barrier function, declines with mutations or reduced expression, increasing dryness and environmental vulnerability [16]. Additional pathways, including NRF2/HO‐1, NF‐κB [17], EGFR [18], and MEK/ERK [19], also regulate structural and functional changes in aging skin.

Traditional Chinese Medicine (TCM) excels in addressing complex diseases through its multi‐component, multi‐target approach, modulating diverse biological pathways [20].

This is advantageous for multifactorial conditions like skin aging. Studies highlight TCM formulations with anti‐aging potential [21, 22, 23], rooted in compatibility theory, which emphasizes herbal synergy. For instance, the refined Qing Kai Ling formula—combining Baicalin, Jasminoidin, and Desoxycholic acid—outperforms individual components in treating ischemic stroke, exemplifying TCM's synergistic principles [24].

Optimizing herbal combinations remains a challenge in TCM research. Traditional knowledge, explored via platforms like the Chinese Medicine Inheritance Computing Platform (V3.0) [25], guides formulation. However, for plant resources lacking such heritage—like Cymbidium species, whose by‐products inhibit tyrosinase, elastase, and collagenase [26]—modern pharmacodynamic methods are essential. Selecting compatible herbs to enhance their therapeutic value is a critical research focus.

Network medicine and pharmacology integrate systems biology, genomics, and proteomics to decode drug mechanisms through computational and experimental approaches [27]. This aligns with TCM's holistic nature [27]. This aligns with TCM's holistic nature [28], making it ideal for studying anti‐aging formulations. For example, network pharmacology revealed *Pogostemon cablin* Benth's anti‐aging effects via active ingredients and aging‐related targets [29]. By analyzing drug–disease target interactions, network medicine also aids in screening herbs and designing synergistic prescriptions [30].

This study leverages *Cymbidium kanran* rhizome extract (CKE), known for anti‐skin aging properties, as a case study. A network medicine framework identifies TCM herbs compatible with CKE to create a novel formulation. Network pharmacology elucidates its efficacy and mechanisms, validated through in vitro experiments and clinical trials. We aim to develop an effective anti‐skin aging prescription, confirm its pharmacological basis, and establish a model for formulating herbs lacking traditional knowledge.

## 2 Materials and Methods

### 2.1 Network Medicine Framework Analysis

#### 2.1.1 Collection and Screening of Traditional Chinese Medicine Ingredients and Their Target Genes

Chemical constituents of common Traditional Chinese Medicines (TCM) and their target genes were retrieved from the Traditional Chinese Medicine Systems Pharmacology Database (TCMSP, https://old.tcmsp‐e.com/tcmsp.php) [31]. Active ingredients were defined as those with a drug‐likeness (DL) score > 0.5. To exclude ubiquitous components, constituents present in > 10% of TCMSP herbs were omitted. Given variability in constituent and target numbers across herbs, we capped targets per herb at 20, prioritizing experimentally validated targets, followed by those with high SVM and RF scores. For similar scores, targets with lower frequency across herbs were selected. Target protein gene names were retrieved from UniProt (https://www.uniprot.org/, organism: Human), and Gene IDs were obtained from the NCBI database [32].

For herbs absent from TCMSP, targets were predicted using SuperPred (https://prediction.charite.de/subpages/target_prediction.php) [33], prioritizing validated targets and selecting those with the highest probability, up to a maximum of 20 per herb.

#### 2.1.2 Network‐Based Proximity and Separation

Network‐based proximity (*Z*_dc_) assesses drug‐disease efficacy, while separation (*S*_AB_) evaluates drug synergy within the human interactome [34, 35]. *Z*_dc_ ≤ −0.15 indicates efficacy, and *S*_AB_ ≥ 0 suggests synergy [30]. The computational procedures employed in the network medicine framework were primarily based on the methodology described by Morselli Gysi et al. [30], which provides detailed step‐by‐step protocols and the corresponding source code. The protein–protein interaction (PPI) data required for the calculations were also derived from that same reference, having been compiled and curated from 21 public databases. For disease‐related targets, a set of core targets associated with skin aging was defined, comprising COL1A1, COL1A2, COL3A1, COL4A1, COL4A2, COL4A3, COL4A4, COL4A5, COL7A1, COL10A1, ITGA1, FN1, MMP1, and FLG. Regarding the herbal ingredients, their target profiles were sourced from the TCMSP database or, where applicable, from SuperPred. For each herb, the top 20 targets with the highest predictive scores were retained.

### 2.2 Network Pharmacology‐Based Target Prediction and Pathway Enrichment

This section presents the results of the network pharmacology analysis, which were obtained through computational (*in silico*) approaches. No experimental samples were involved in this part of the study.

#### 2.2.1 Collection of ZYS Ingredients and Targets

The screened formula, named ZiYuShuang (ZYS), had its characteristic components identified based on high content as per the Chinese Pharmacopoeia (2020) or literature (Table 1) [36, 37, 38]. Structures were drawn in ChemBioDraw Ultra 14.0, converted to SMILES, and targets predicted using SwissTargetPrediction (http://www.swisstargetprediction.ch/) was employed to predict targets for these ingredients [39]. For SMILES strings > 200 characters, NetInfer (http://lmmd.ecust.edu.cn/netinfer/) was used [40].

**TABLE 1:** The representative ingredients of ZYS.

Herb name	Compound name	No
*Cymbidium kanran* rhizome extract	Adenosine	ZY1
	Biotin	ZY2
	Choline	ZY3
*Panax ginseng* root extract	Ginsenoside Rg1	ZY4
	Ginsenoside Rb1	ZY5
	Ginsenoside Re	ZY6
*Ganoderma atrum* extract	Ganoderic acid A	ZY7
	Lucidenic acid A	ZY8
	Ganoderic acid H	ZY9
*Polygonatum cyrtonema* rhizome/root extract	Yamogenin	ZY10
	Quercetin	ZY11
	Kaempferol	ZY12
*Rehmannia glutinosa* Radix Praeparata extract	Rehmannioside D	ZY13
	Verbascoside	ZY14
	Catalpol	ZY15

#### 2.2.2 Screening Skin Aging Targets

Skin aging targets were sourced from GeneCards (https://www.genecards.org/) using keywords “oxidative stress”, “inflammation”, and “apoptosis” [41], supplemented by literature‐derived targets. Duplicates and targets with scores < 20 were removed.

#### 2.2.3 Screening ZYS Anti‐Skin Aging Targets

Intersection analysis identified overlapping targets between ZYS components and skin aging targets as potential anti‐aging targets. Molecular docking assessed affinity between ZYS components and key skin aging targets (Table 2), including targets with affinity < −5 kcal/mol. Docking involved: drawing ZYS structures in ChemDraw, energy minimization in Chem3D, conversion to mol2 and pdbqt formats in AutoDock Tools [42], retrieving protein structures from PDB, and removing water, ligands, and ions in PyMOL 3.1 [43]. AutoDock Tools added hydrogens and charges, DeepSite identified docking sites, and AutoDock Vina performed docking [42, 44].

**TABLE 2:** The information of targets used in autodock analysis.

Gene symbol	Target name	PDB ID
COL1A1	Collagen alpha‐1(I) chain	3EJH
COL1A2	Collagen alpha‐2(I) chain	5CTD
COL3A1	Collagen alpha‐1(III) chain	4AE2
COL4A1	Collagen alpha‐1(IV) chain	1LI1
COL4A3	Collagen alpha‐3(IV) chain	6WKU
COL7A1^a^	Collagen alpha‐1(VII) chain	6S4C
COL10A1^b^	Collagen alpha‐1(X) chain	
ITGA1	Integrin alpha‐1	1PT6
ITGA2	Integrin alpha‐2	1AOX
ITGA4	Integrin alpha‐4	3V4P
ITGA5	Integrin alpha‐5	4WJK
ITGA6	Integrin alpha‐6	7CEB
ITGB1	Integrin beta‐1	3G9W
ITGB2	Integrin beta‐2	1YUK
ITGB3	Integrin beta‐3	1JV2
ITGB4	Integrin beta‐4	3F7P
ITGB5	Integrin beta‐5	7S46
ITGB6	Integrin beta‐6	4UM8
ITGB7	Integrin beta‐7	3V4P
ITGB8	Integrin beta‐8	6OM1
FN1	Fibronectin	1FNH
MMP1	22 kDa interstitial collagenase	1CGL
FLG	Filaggrin	4PCW

^a^ Mouse‐derived proteins.

^b^ Proteins generated by homology modeling method.

#### 2.2.4 GO and KEGG Pathway Enrichment

ZYS anti‐skin aging targets underwent Gene Ontology (GO) biological process and KEGG pathway enrichment analysis using the DAVID database (http://david.abcc.ncifcrf.gov/) [45].

#### 2.2.5 “Ingredients‐Targets‐Pathways” Network Construction

Cytoscape 3.10.0 constructed an “ingredients‐targets‐pathways” network, with nodes as ingredients, targets, and pathways, and edges linking ingredients to targets and pathways [46]. The Analyze Network tool identified key nodes by degree value.

### 2.3 Sample Preparation

The ZiYuShuang (ZYS) was prepared by Shanghai Jahwa United Co. Ltd., combining *Cymbidium kanran* rhizome extract (CKE), *Panax ginseng* root extract, *Rehmannia glutinosa* Radix Praeparata extract, *Ganoderma atrum* extract and *Polygonatum cyrtonema* rhizome/root extract with excipients, as previously report [23]. SZS was similarly prepared, excluding *Rehmannia glutinosa* and *Polygonatum cyrtonema* extracts. All herbal extracts were prepared using water as the extraction solvent. Excipients included 1,2‐hexanediol, ammonium acryloyldimethyltaurate/VP copolymer, aqua, behenyl alcohol, betaine, butylene glycol, caprylic/capric triglyceride, carbomer, carnosine, cetearyl alcohol, cetearyl olivate, CI 17200, CI 47005, citric acid, collagen amino acids, dicaprylyl carbonate, diisostearyl malate, dimethicone, dipeptide diaminobutyroyl benzylamide diacetate, disodium EDTA, ectoin, glycereth‐26, glycerin, hydrogenated lecithin, hydroxyacetophenone, jojoba esters, niacinamide, palmitoyl tetrapeptide‐7, palmitoyl tripeptide‐1, PEG‐40 hydrogenated castor oil, pentaerythrityl tetraethylhexanoate, phenoxyethanol, polyglyceryl‐10 myristate, polyquaternium‐51, polysorbate 20, saccharide isomerate, sodium chloride, sodium citrate, sodium lactate, sodium sulfate, sorbitan olivate, and squalane.

### 2.4 Experimental Validation

#### 2.4.1 UVA‐Induced Human Skin Fibroblasts

Human dermal fibroblasts derived from the skin of an 8‐ to 10‐year‐old donor (Guangdong Biocell Biotechnology Co. Ltd.) were cultured overnight in 6‐well plates to 60% confluence under standard conditions (37°C, 5% CO_2_). At 40%–50% confluence, drugs were added in triplicate: blank and negative controls received 2 mL culture medium, positive control received medium with TGF‐β1, and sample groups received medium with test samples. After 24‐h incubation (37°C, 5% CO_2_), all groups except the blank underwent 30 mJ/cm^2^ UVA exposure, followed by another 24‐h incubation. In this experiment, the tested groups included a blank control group (BC), a negative control group (NC), positive control groups (PC and PXY), and three treatment groups (CKE, SZS, and ZYS). For the positive controls, PC was treated with TGF‐β1 at a concentration of 100 ng/mL, while PXY received Pro‐Xylane at 0.5 mg/mL. The treatment groups received extracts at a final concentration of 0.04%, corresponding to their respective formulation concentrations. The same group setup was applied to all subsequent experiments.

#### 2.4.2 H_2_O_2_ ‐Induced Human Skin Fibroblasts

Fibroblasts at 60% confluence were seeded in 6‐well plates and incubated overnight (37°C, 5% CO_2_). At 40%–60% confluence, all groups except the blank were treated with H_2_O_2_ for 2 h. Drugs were then added in triplicate: blank and negative controls received 2 mL medium, positive control received medium with TGF‐β1, and sample groups received medium with test samples. Plates were incubated for 22 h (37°C, 5% CO_2_), repeated thrice. Cells were digested, reseeded, and incubated for 24 h. Medium was removed, cells were washed with PBS, fixed with 1 mL β‐galactosidase fixative (15 min, room temperature), washed thrice with PBS, stained with 1 mL staining solution, and incubated overnight (37°C). Plates were sealed and examined microscopically.

#### 2.4.3 Quantitative Reverse Transcription PCR (RT‐qPCR)

RNA was extracted from fibroblasts using RNA iso Plus Reagent, and 1 μg mRNA was reverse‐transcribed into cDNA with the PrimeScript RT Reagent Kit. Elastin, collagen types I, III, IV, VII, and decorin mRNA levels were quantified via qPCR with SYBR Green on a Bio‐Rad CFX96.

#### 2.4.4 Enzyme‐Linked Immunosorbent Assay (ELISA)

MMP‐1 release in UVA‐irradiated fibroblasts was quantified using an ELISA kit (R&D Systems Inc., Minneapolis, MN, USA), following the manufacturer's protocol.

#### 2.4.5 Western Blot Analysis

Fibroblasts were seeded at 2 × 10^5^ cells/well in 6‐well plates. At 60%–70% confluence, drugs were added, and cells were cultured for > 48 h. Cells were lysed (20 min), centrifuged (10 000 rpm, 15 min, 4°C), and supernatant protein was quantified with a BCA kit. Samples (50 μg) underwent electrophoresis (120 V, 1 h), transfer to PVDF (300 mA, 65 min), blocking (1 h), overnight primary antibody incubation (4°C), TBST washing, and secondary antibody incubation (1 h). Collagen types III, IV, and β‐actin bands were developed and imaged.

### 2.5 Open Prospective, Evaluator‐Blinded Trial of ZYS

#### 2.5.1 Subjects

Participants were healthy Chinese females aged 30–55 (*n* = 33, mean age 43.48 ± 7.39 years) meeting inclusion criteria: voluntary participation with informed consent, facial TEWL ≥ 15 g/h m^2^, willingness to maintain a regular lifestyle, and ability to complete questionnaires. Exclusion criteria included allergies, severe skin conditions, facial marks affecting results, recent skin treatments, active systemic diseases, steroid/immunosuppressant use, pregnancy/breastfeeding, recent trial participation, or employment in cosmetics.

#### 2.5.2 Study Design

This open, prospective, evaluator‐blinded trial involved written consent and suitability screening. Participants avoided cosmetics the day prior to testing, conducted at 20°C–22°C and 40%–60% humidity. After 30‐min acclimation, assessments occurred pre‐application, 15 min post‐application, and at 8 h, 2 weeks, and 4 weeks. ZYS was applied evenings to the face, eye area, and neck with gentle massage. The protocol and consent form were approved on 20 December 2023 by the Ethics Committee of Shanghai Institute of Spice Research Co. Ltd. (2023‐LLSC‐GP231115~1121).

#### 2.5.3 Efficacy Assessment

Efficacy was evaluated per Table 3: **(1) Skin hydration:** Measured with a Corneometer (Courage & Khazaka, Germany) at the left outer eye corner–nose junction (3 readings averaged, C.U.; higher = greater hydration). **(2) Transepidermal water loss (TEWL):** Assessed with a Tewameter TM 300 (Courage & Khazaka) at the same site (30 s, last 20 s averaged, g/h m^2^; lower = better barrier). **(3) Skin coloration:** Measured with a CM‐700d spectrophotometer (Konica Minolta, Japan) for a* (redness) and b* (yellowness) (3 readings averaged, a.u.; lower = less redness/yellowness). **(4) Skin luminosity:** Assessed with a Skin‐Glossymeter GL200 (right outer eye corner–nose, 3 readings averaged, a.u.; higher = greater radiance). **(5) Skin firmness and elasticity:** Measured with a Cutometer (right outer eye corner–nose, 3 readings averaged, a.u.; R2/R7 near 1 = better elasticity/recovery, lower R0 = tighter skin). **(6) Skin translucency:** Assessed with a TLS 850 (left cheek, a.u.; lower ALPHA, higher K/AREA = better transparency). **(7) Skin density and thickness:** Measured with a DermaLabUSB (Cortex, Denmark) at the left eye midline–nose intersection (density in a.u., higher = better; thickness in μm, higher = thicker). **(8) Skin texture:** Evaluated with a VC20 plus microscope and SELS system (left outer eye corner–nose, 3 images averaged; lower SER/SESC/SEW = less roughness/flakiness/wrinkles, higher SESM = smoother). **(9) Imaging assessment:** VISIA‐CR (Canfield Scientific Inc., USA) captured full/left/right face images under standard, cross‐polarized, and parallel‐polarized light. Image Pro Plus 7.0 analyzed jawline angle (a.u., smaller = tighter) and forehead lines, nasolabial folds, and crow's feet (mm^2^ and count, smaller/fewer = less visible). **(10) Visual assessment:** Evaluators rated facial lines (forehead, glabellar, nasolabial, crow's feet, marionette) using a VAS (0–10, 0 = inconspicuous, 9–10 = very pronounced) and skin aging chart.

**TABLE 3:** Lists the efficacy evaluation criteria for ZYS at each test time point.

Evaluation criteria	Before‐application	Post‐application			
	0 min (BA)	15 min (PA‐15 min)	8 h (PA‐8 h)	2 weeks (PA‐2 weeks)	4 weeks (PA‐4 weeks)
Skin hydration	√	√	√	√	√
Transepidermal water loss	√	×	√	√	√
Skin coloration	√	√	√	√	√
Skin luminosity	√	√	√	√	√
Skin firmness and elasticity	√	×	√	√	√
Skin translucency	√	√	√	√	√
Skin density and thickness	√	×	×	√	√
Skin texture	√	√	√	√	√
Imaging assessment	√	√	√	√	√
Visual assessment	√	×	√	√	√

Abbreviations: √, requires testing; ×, does not require testing.

#### 2.5.4 Statistical Analysis

Data were analyzed in SPSS. Normality was tested for continuous data; paired *t*‐tests were used for normally distributed paired data, and Wilcoxon signed‐rank tests for non‐normal or ordinal data (*p* < 0.05). Bar charts were generated in GraphPad Prism 9.

## 3 Results

### 3.1 Screened Anti‐Skin Aging Formula

From the TCMSP database, 427 herbal medicines were extracted and classified into 38 groups by medicinal properties. Seventeen categories with ≥ 10 herbs, termed large varieties of traditional Chinese medicines (LVTCM), comprised 80.3% (343 herbs), including Antipyretic Detoxification Drugs (64 herbs), Diuretic Dampness‐Excreting Drugs (32), Blood Activating Stasis‐Removing Drugs (27), Phlegm Resolving Medicine (26), and Qi Regulating Drugs (25). Small varieties (SVTCM), spanning 21 categories, accounted for 19.7% (84 herbs). *Z*‐values for skin aging were calculated, identifying 155 herbs (36.3%) with *Z* ≤ −0.15, indicating potential anti‐skin aging activity (Table S1). LVTCM included 123 active herbs (35.9%), while SVTCM had 28 (38.1%). Variability was higher in SVTCM due to smaller sample sizes per category, increasing randomness in identifying active herbs.

Among LVTCM, seven categories exceeded 40% active herbs: Qi Reinforcing Drugs (53.33%), Pungent Cool Diaphoretics (53.85%), Yang Reinforcing Drugs (46.67%), Phlegm Resolving Medicine (42.31%), Wind‐Dampness Dispelling and Cold‐Dispersing Medicinals (47.83%), Qi Regulating Drugs (48.00%), and Antitussive Antiasthmatics (41.18%). Qi Reinforcing Drugs (mean *Z* = −0.23 ± 0.95) and Pungent Cool Diaphoretics (−0.22 ± 0.65) showed the strongest potential. Of 37 herbs with *Z* < −1, 19 were cosmetic candidates, including Ganoderma, *Ginseng* Radix et Rhizoma, and *Notoginseng* Radix et Rhizoma. Qi Reinforcing Drugs and Blood Activating Stasis‐Removing Drugs each had three highly active herbs, suggesting a link between quantity and efficacy in Qi Reinforcing Drugs. Thus, we prioritized this category for synergy with *Cymbidium kanran*.

From Qi Reinforcing Drugs, Ganoderma, *Notoginseng* Radix et Rhizoma, and *Ginseng* Radix et Rhizoma exhibited the strongest activity. Due to chemical similarity between Notoginseng and Ginseng, we combined Ganoderma and *Ginseng* Radix et Rhizoma with *Cymbidium kanran*, yielding separation values (*S*_AB_) of 0.23 and 0.13, respectively, indicating synergy. This trio, dubbed “Sanzhen”, was expanded with *Rehmanniae* Radix Praeparata (*Z* = −0.22) and *Polygonati Rhizoma*. Initially, *Polygonati Rhizoma*'s *Z* = 0.9 suggested inactivity, contradicting prior data [47]. Recalculating with quercetin and kaempferol as key actives yielded *Z* = −2.20, confirming its efficacy. Sanzhen plus Rehmanniae Radix Praeparata‐Polygonati Rhizoma (*S*_AB_ = 0.05) showed synergy, forming “ZiyuShuang” (ZYS).

### 3.2 Network Pharmacology‐Based Target Prediction and Pathway Enrichment

#### 3.2.1 Skin Aging Disease Targets

Screening GeneCards identified 156 oxidative stress, 17 inflammation, and 52 apoptosis targets (score > 20), totaling 205 unique targets. Literature added 114 more, yielding 319 skin aging targets (Table S2).

#### 3.2.2 ZYS Anti‐Skin Aging Targets

SwissTargetPrediction and NetInfer identified 631 ZYS targets. Molecular docking (Table S3) showed affinities < −5 kcal/mol for all components except ZY3, indicating strong binding to skin aging targets. Intersection analysis with skin aging targets revealed 96 anti‐aging targets for ZYS.

#### 3.2.3 GO and KEGG Enrichment

**FIGURE 1:**
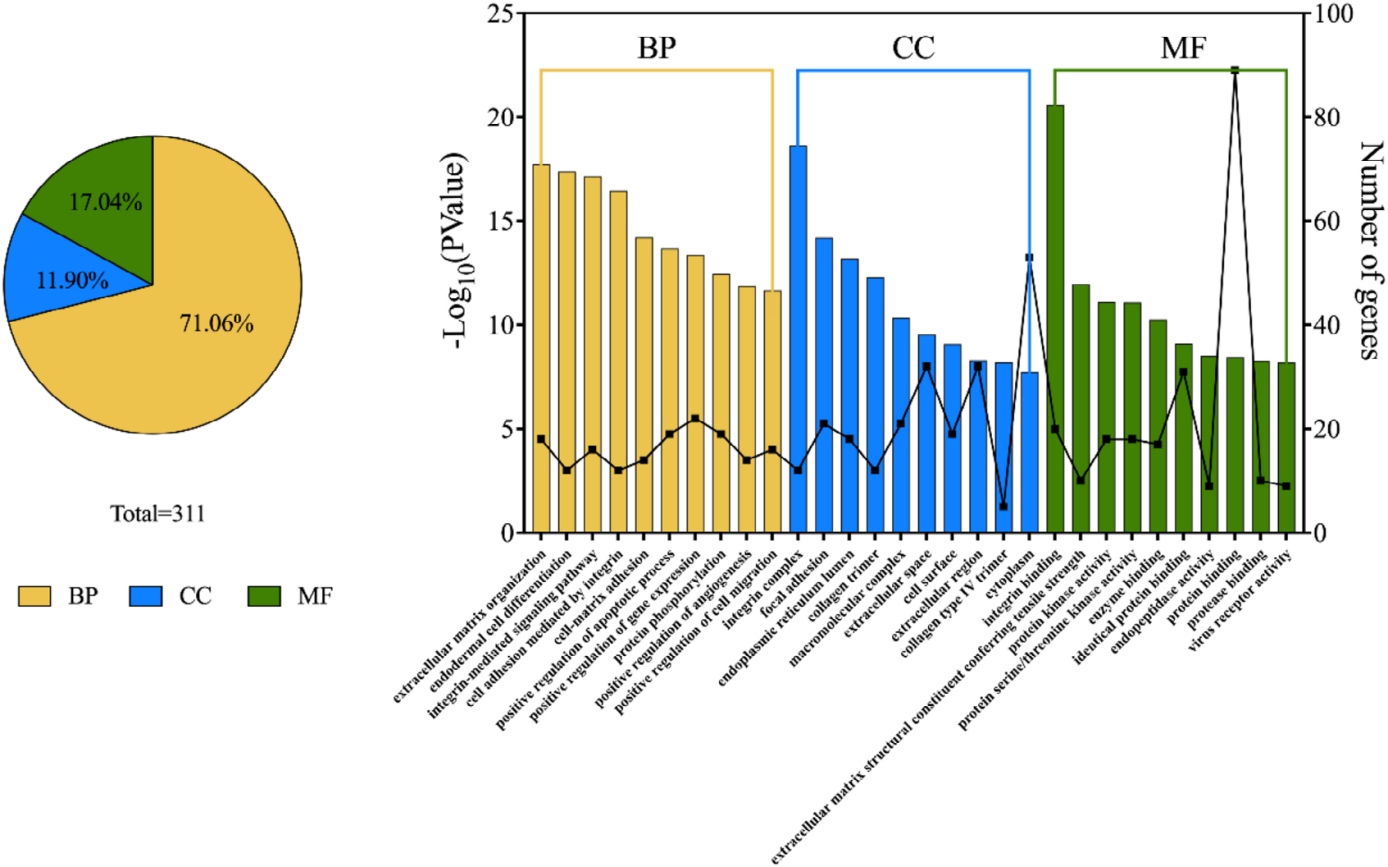
GO enrichment analysis for the anti‐skin aging targets of ZYS. The order of importance was ranked from left to right by −log10 (*p*‐value) with a bar chart. The number of targets sticks into each term with a line chart. BP, biological process; CC, cell composition; GO, gene ontology; MF, molecular function.

**FIGURE 2:**
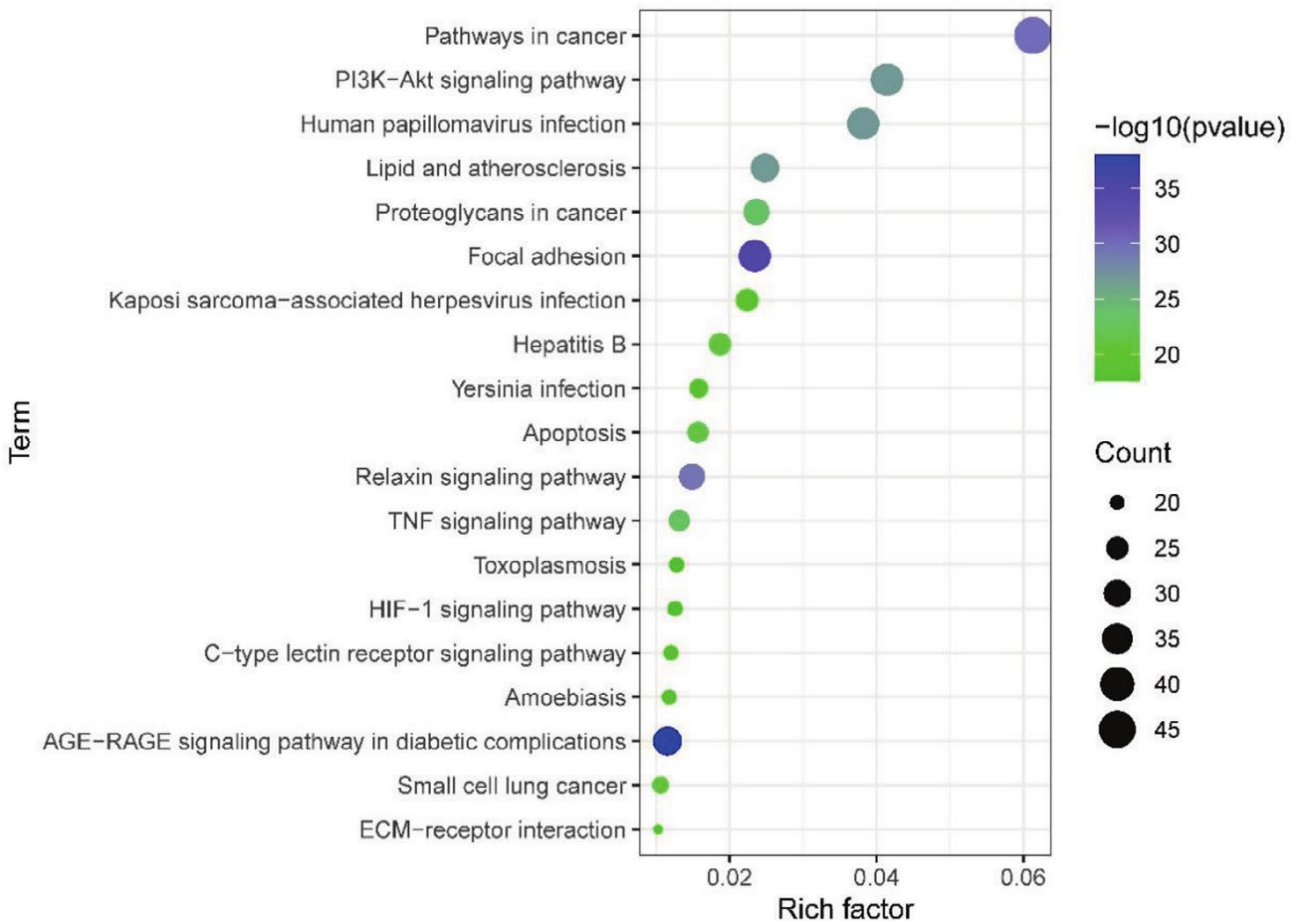
KEGG pathway enriched by the anti‐skin aging targets of ZYS. The size of the bubbles in each bubble chart represents the gene counts in each pathway and the colors from green to blue represent the *p*‐values from small to large.

GO analysis of 96 ZYS targets (*p* < 0.01) identified 221 biological processes (BP), 37 cellular components (CC), and 53 molecular functions (MF). Top BPs included extracellular matrix organization and integrin‐mediated signaling; top CCs were integrin complex and focal adhesion; top MFs included integrin binding and protein kinase activity (Figure 1). KEGG analysis identified 150 pathways (*p* < 0.01): 70 disease‐related (e.g., AGE‐RAGE signaling, cancer pathways) and 80 others (e.g., focal adhesion, PI3K‐Akt, TNF signaling, apoptosis) (Figure 2).

#### 3.2.4 Ingredients‐Targets‐Pathways Network

**FIGURE 3:**
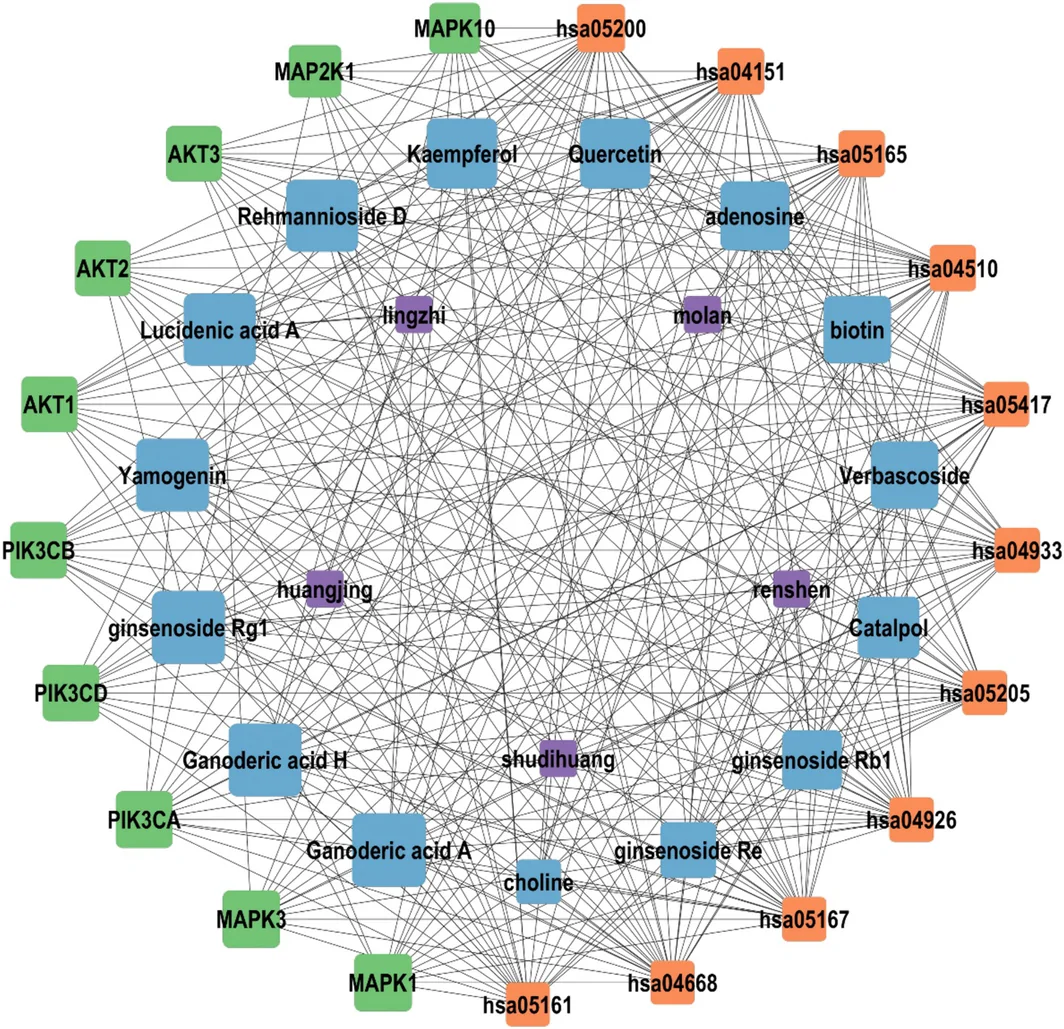
Network of the main ingredients, pathways and targets of ZYS for anti‐skin aging. Nodes represent herbs, ingredients, targets and pathways were filled with Purple, blue, green, and orange, respectively.

The ZYS network (Cytoscape, Figure S1) comprised 267 nodes (herbs, ingredients, targets, pathways) and 4668 edges. Top targets by degree were MAPK1, MAPK3, PIK3CA, PIK3CB, PIK3CD, AKT1, AKT2, AKT3, MAP2K1, and MAPK10; top pathways included PI3K‐Akt, focal adhesion, and relaxin signaling (Table 4 and Figure 3).

**TABLE 4:** The top 10 pathways and targets in ingredients‐targets‐pathways network.

Pathway name	Degree	Name	Degree
PI3K‐Akt signaling pathway	53	MAPK1	111
Focal adhesion	51	MAPK3	110
Relaxin signaling pathway	43	PIK3CA	107
Regulation of actin cytoskeleton	37	PIK3CB	105
Apoptosis	37	PIK3CD	105
HIF‐1 signaling pathway	34	AKT1	102
ECM‐receptor interaction	33	AKT2	100
C‐type lectin receptor signaling pathway	33	AKT3	100
IL‐17 signaling pathway	32	MAP2K1	85
Rap1 signaling pathway, FoxO signaling pathway, Estrogen signaling pathway	31	MAPK10	75

### 3.3 Experimental Validation

**FIGURE 4:**
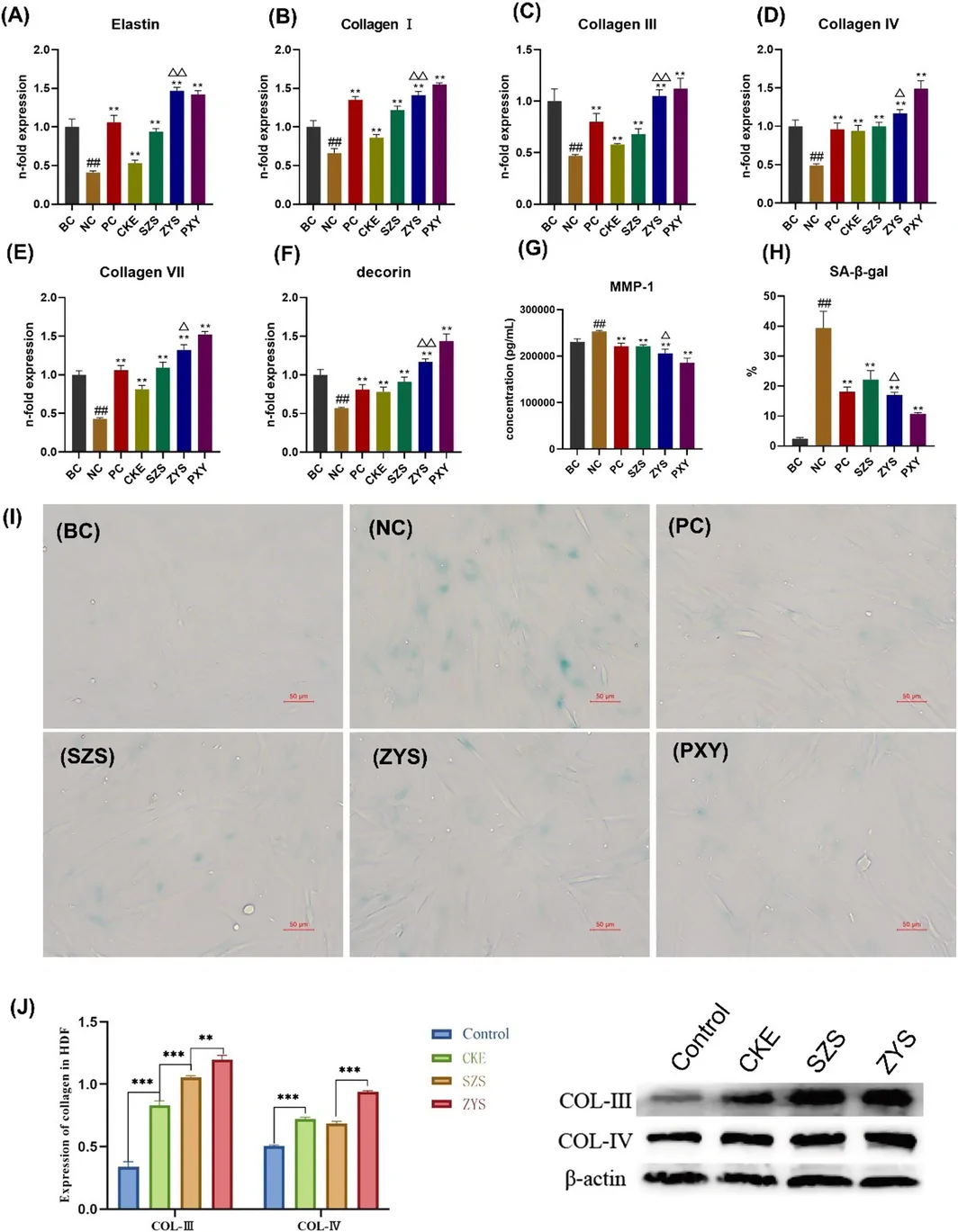
The impact of CKE, SZS, and ZYS on the alterations in the levels of Elastin (A), Collagen I (B), Collagen III (C), Collagen IV (D), Collagen VII (E), decorin (F) and MMP‐1 (G) in UVA‐induced human skin fibroblast cells. The influence of CKE, SZS, and ZYS on the content changes of SA‐β‐gal in H2O2‐induced human skin fibroblast cells detected by staining (H, I). Statistical significance in panels is represented by: #(*p* < 0.05) and ##(*p* < 0.01) vs. BC; *(*p* < 0.05) and **(*p* < 0.01) vs. NC; ∆(*p* < 0.05) and ∆∆(*p* < 0.01) vs. SZS. The effects of CKE, SZS, and ZYS on the content changes of Collagen III and Collagen IV in Fibroblast cells by western blot (J). Statistical significance in panels is represented by: **(*p* < 0.05) and ***(*p* < 0.01).

UVA‐irradiated fibroblasts treated with CKE, SZS, and ZYS showed upregulated elastin (Figure 4A), collagen IV (Figure 4D), and collagen VII (Figure 4E) gene expression versus controls. CKE increased these by 30.01% ± 5.29%, 92.37% ± 6.29%, and 88.55% ± 13.75%; SZS by 130.44% ± 14.55%, 104.21% ± 10.58%, and 153.31% ± 11.78%; ZYS by 258.75% ± 6.98%, 139.15% ± 17.75%, and 207.63% ± 9.14%; and PXY (positive control) by 245.72% ± 7.47%, 202.71% ± 17.01%, and 253.67% ± 15.66%. ZYS matched PXY for elastin and outperformed SZS and CKE (*p* < 0.05). For collagen I (Figure 4B), III (Figure 4C), and decorin (Figure 4F), CKE upregulated by 32.27% ± 17.04%, 23.64% ± 4.76%, and 36.78% ± 9.46%; SZS by 86.48% ± 21.32%, 45.23% ± 14.29%, and 59.70% ± 10.11%; ZYS by 114.64% ± 18.70%, 124.27% ± 10.89%, and 105.30% ± 8.12%; and PXY by 136.48% ± 20.38%, 140.61% ± 18.51%, and 152.87% ± 21.00%. Western blotting confirmed collagen III (CKE: 148.28% ± 21.24%, SZS: 216.49% ± 43.84%, ZYS: 259.33% ± 47.55%) and IV (CKE: 43.03% ± 1.79%, SZS: 35.75% ± 1.64%, ZYS: 86.76% ± 1.09%) increases (Figure 4J). ZYS inhibited MMP‐1 by 18.59% ± 2.85% vs. SZS (12.62% ± 1.96%) and PXY (26.57% ± 3.26%) (Figure 4G). In H_2_O_2_‐treated fibroblasts, ZYS reduced SA‐β‐gal‐positive cells by 56.45% ± 7.63% vs. SZS (43.07% ± 10.85%) and PXY (72.83% ± 3.33%) (Figure 4H,I), indicating anti‐wrinkle efficacy.

### 3.4 Clinical Effectiveness

**FIGURE 5:**
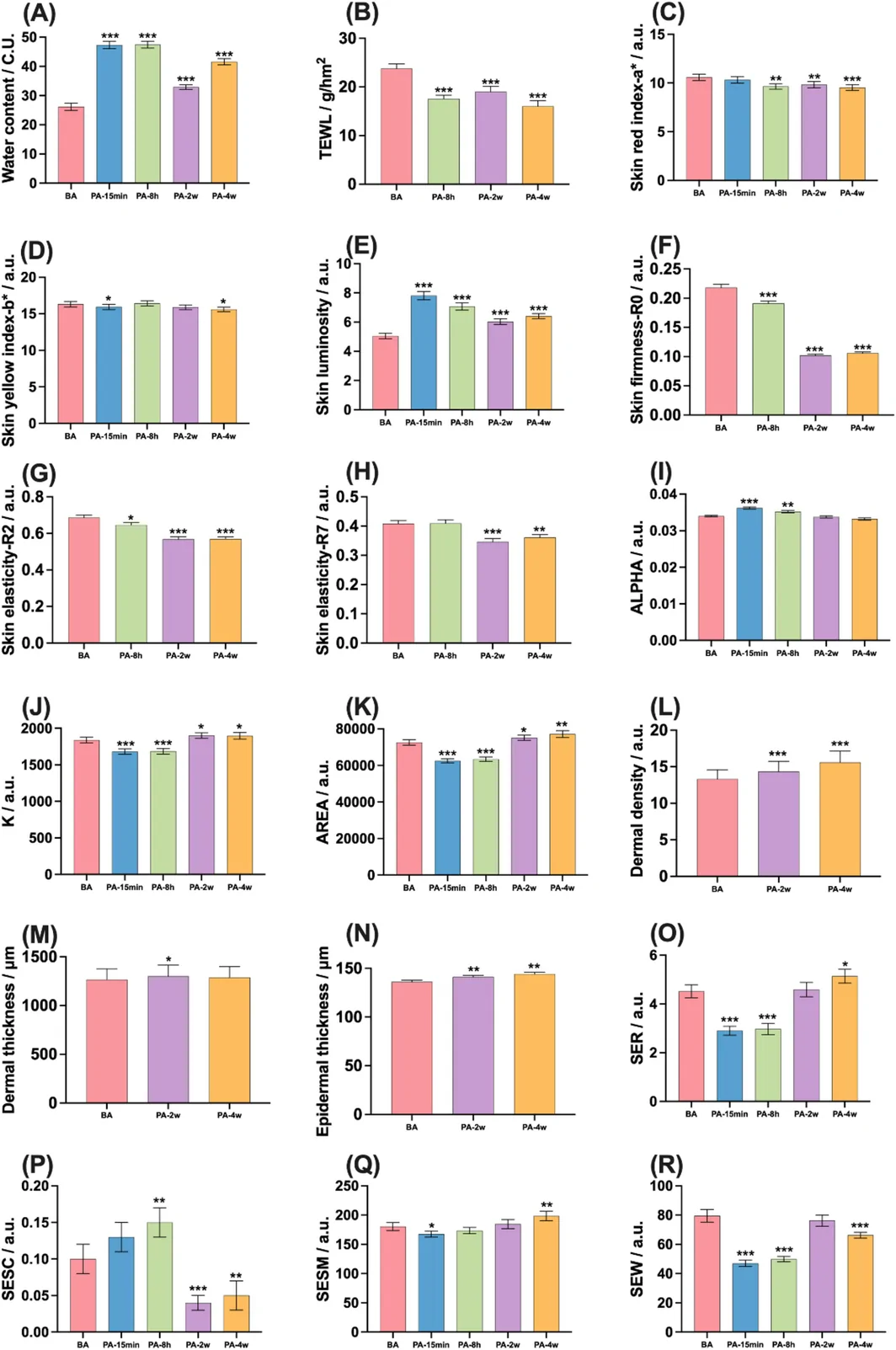
The clinical efficacy of ZYS in anti‐aging skin treatment assessed through instrument analysis. (A) Skin hydration. (B) Transepidermal water loss (TEWL). (C) Skin red index‐a*. (D) Skin yellow index‐b*. (E) Skin luminosity. (F) Skin firmness‐R0. (G) Skin elasticity‐R2. (H) Skin elasticity‐R7. (I) Skin translucency‐ALPHA. (J) Skin translucency‐K. (K) Skin translucency‐AREA. (L) Dermal density. (M) Dermal thickness. (N) Epidermal thickness. (O) Skin roughness (SER). (P) Skin flakiness (SESC). (Q) Skin smoothness (SESM). (R) Skin wrinkle (SEW). (*p < 0.05, **p < 0.01, ***p < 0.001).

**FIGURE 6:**
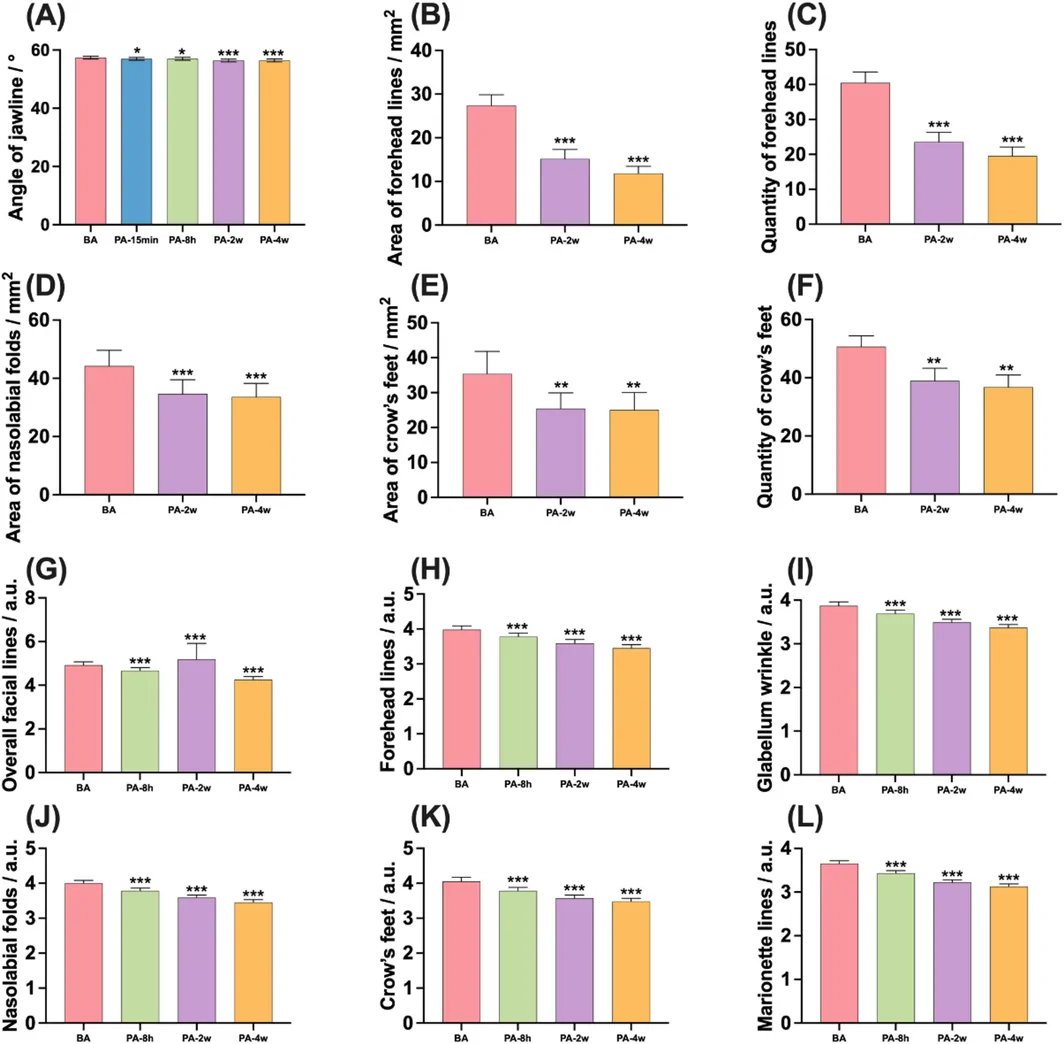
The clinical efficacy of ZYS in anti‐aging skin treatment evaluated through imaging assessment (A–F) and visual assessment (G–L). (A) Angle of jawline. (B) Area of forehead lines. (C) Quantity of forehead lines. (D) Area of nasolabial folds. (E) Area of crow's feet. (F) Quantity of crow's feet. (G) Overall facial lines. (H) Forehead lines. (I) Glabellum wrinkle. (J) Nasolabial folds. (K) Crow's feet. (L) Marionette lines. (*p < 0.05, **p < 0.01, ***p < 0.001).

Baseline skin hydration was 26.17 ± 1.26 C.U., increasing by 80.87% (15 min), 81.33% (8 h), 25.83% (2 weeks), and 59.00% (4 weeks) (*p* < 0.001) (Figure 5A). TEWL (23.76 ± 1.00 g/h·m^2^) decreased by 26.22% (8 h), 19.96% (2 weeks), and 32.63% (4 weeks) (*p* < 0.001) (Figure 5B). Redness (a*, 10.58 ± 0.32 a.u.) dropped by 8.76% (8 h, *p* < 0.01), 7.05% (2 weeks, *p* < 0.01), and 9.93% (4 weeks, *p* < 0.001) (Figure 5C); yellowness (b*, 16.30 ± 0.38 a.u.) by 2.20% (immediate, *p* < 0.05) and 4.36% (4 weeks, *p* < 0.05) (Figure 5D). Glossiness (5.05 ± 0.19 a.u.) rose by 54.54% (immediate), 39.60% (8 h), 19.40% (2 weeks), and 26.77% (4 weeks) (*p* < 0.001) (Figure 5E). Firmness (R0, 0.218 ± 0.006 a.u.) decreased by 12.68% (8 h), 53.44% (2 weeks), and 51.44% (4 weeks) (*p* < 0.001) (Figure 5G). Elasticity (R2, 0.686 ± 0.014 a.u.; R7, 0.408 ± 0.011 a.u.) improved significantly (*p* < 0.05) (Figure 5H). Translucency (ALPHA, K, AREA) increased immediately and at 8 h (*p* < 0.01) (Figure 5I–K). Dermal density (13.30 ± 1.27 a.u.) rose by 7.81% (2 weeks) and 17.24% (4 weeks) (*p* < 0.001) (Figure 5L); dermal thickness (1265 ± 112.52 μm) by 2.82% (2 weeks, *p* < 0.05) (Figure 5M); epidermal thickness (136 ± 1.67 μm) by 3.49% (2 weeks) and 5.63% (4 weeks) (*p* < 0.01) (Figure 5N). Roughness (SER, 4.52 ± 0.27 a.u.) dropped by 35.86% (immediate) and 34.37% (8 h) (*p* < 0.001) (Figure 5O); flakiness (SESC, 0.10 ± 0.02 a.u.) by 64.71% (2 weeks, *p* < 0.001) and 50.10% (4 weeks, *p* < 0.01) (Figure 5P); wrinkles (SEW, 79.53 ± 4.34 a.u.) by 40.92% (immediate), 37.28% (8 h), and 16.69% (4 weeks) (*p* < 0.01) (Figure 5R). Jawline angle (57.37° ± 0.50°) reduced by 0.70%–1.70% (*p* < 0.05) (Figure 6A); forehead lines (area: 27.34 ± 2.52 mm^2^, count: 40.48 ± 3.14) by 44.56%–56.76% and 41.77%–51.65% (*p* < 0.001) (Figure 6B,C); nasolabial folds (44.24 ± 5.37 mm^2^) by 21.76%–23.99% (*p* < 0.001) (Figure 6D); crow's feet (area: 35.37 ± 6.46 mm^2^, count: 50.67 ± 3.75) by 28.26%–29.27% and 23.15%–27.39% (*p* < 0.01) (Figure 6E,F). Overall facial lines (4.92 ± 0.15 a.u.) decreased at 8 h and 4 weeks (*p* < 0.001) (Figure 6G). No irritation or allergies were reported.

## 4 Discussion

Beyond intrinsic aging, skin, as the body's outermost barrier, is highly susceptible to UV‐induced oxidative stress, accelerating photoaging [48]. ZiyuShuang (ZYS), a five‐herb formula (*Cymbidium kanran* rhizome extract (CKE), *Panax ginseng* root extract, *Rehmannia glutinosa* Radix Praeparata extract, *Ganoderma atrum* extract, and *Polygonatum cyrtonema* rhizome/root extract), is proposed for anti‐aging. Its components reportedly modulate oxidative stress, inflammation, and apoptosis, suggesting potential to delay photoaging and enhance rejuvenation [49, 50, 51, 52]. This study integrates clinical, network pharmacology, and in vitro approaches to clarify ZYS's mechanisms.

Over 4 weeks, ZYS improved photodamage markers—hydration, TEWL, coloration, luminosity, firmness, elasticity, density, thickness, jawline angle, and facial lines. Translucency improved only after 2 weeks, while texture enhanced short‐term. Skin dryness, tied to stratum corneum water‐lipid imbalance [53], is a key aging sign often targeted by cosmetics or supplements [54]. Increased density and thickness likely reduced TEWL, a primary mechanism for ZYS's efficacy.

UVB activates MAPK pathways (*p*38, ERK, JNK) in skin cells, offering photoprotective targets [55]. The PI3K/Akt pathway regulates growth, differentiation, and homeostasis, with therapeutic potential in dermatology [56]. ZYS's core targets—MAPK1, MAPK3, PIK3CA–PIK3CD, AKT1–AKT3, MAP2K1, MAPK10—align with MAPK and PI3K/Akt pathways, suggesting these mediate its anti‐aging effects. MAPK influences transcription factors (Elk‐1, CREB, c‐Fos, c‐Jun), forming AP‐1 to activate MMPs and collagenases, degrading collagen and elastin [57]. Active compounds like Ginsenoside Rk1 [58] and Esculetin [59] enhance collagen via these pathways.

In ZYS's network, MAPK and PI3K/Akt dominate due to broad signaling roles, overshadowing direct targets like elastin and collagen. Yet, docking confirms strong binding, and in vitro data show ZYS upregulates elastin, collagen (I, III, IV, VII), decorin, and MMP‐1, supporting its anti‐aging role. Whether ZYS acts directly or via MAPK/PI3K‐Akt requires further study. Among the 631 targets predicted by network pharmacology for ZYS, we selected elastin, collagens (COL1A1, COL3A1), decorin, and MMP‐1 for in vitro validation based on their central roles in extracellular matrix homeostasis and their frequent identification as hub nodes in our network analysis. However, we acknowledge that numerous other predicted targets (such as MAPK1 and AKT1) remain unvalidated experimentally. These targets may mediate additional anti‐aging pathways, including cell survival, proliferation, and oxidative stress responses. The mechanistic understanding of ZYS remains incomplete, as only a subset of the predicted targets has been experimentally confirmed.

In the present study, we performed a 4‐week open prospective clinical trial involving 33 healthy female participants, measuring a comprehensive set of biophysical parameters—including skin hydration, transepidermal water loss, elasticity, dermal density, thickness, and wrinkle severity. These outcomes inherently capture the integrated biological responses of full‐thickness skin, encompassing both epidermal and dermal compartments. We recognize that three‐dimensional (3D) skin models constitute sophisticated in vitro platforms that closely mimic the structural organization and cellular diversity of native human skin, and they have become instrumental in dermatological research for assessing topical products [60]. Accordingly, deploying such models in the present study could offer additional mechanistic granularity, particularly regarding the distinct roles of various cell populations and dermal matrix turnover. In future investigations, integrating 3D skin models will be valuable for dissecting the candidate pathways emerging from our network pharmacology and in vitro fibroblast data, thus providing a mechanistic bridge to complement the clinical efficacy endpoints.

The most significant limitation of this study is the single‐arm, open‐label design without a placebo or vehicle control group. Therefore, the observed improvements cannot be definitively attributed to ZYS formula alone, as natural skin variations over time or participant expectations may have contributed to the outcomes. To balance feasibility with rigor, only 11 core targets of skin aging were adopted in network medicine analysis. Ideally, if computational resources permit, incorporating all targets from the outset would be preferable. The use of fibroblasts from a young donor (8–10 years old) does not fully recapitulate the cellular characteristics of aged skin. This may limit the translational relevance of our in vitro findings to the geriatric population. Future studies should validate our findings using fibroblasts from older donors (≥ 60 years) and, ideally, primary cells derived from aged human skin biopsies.

## 5 Conclusion

This study evaluated the anti‐skin aging effects of ZiyuShuang (ZYS) through a single‐arm, quasi‐randomized controlled trial integrated with network pharmacology. Results demonstrate that ZYS significantly improves skin hydration, glossiness, translucency, dermal density, and thickness, while substantially reducing redness, yellowness, firmness, roughness, and the extent of forehead lines, nasolabial folds, crow's feet, and overall facial line scores. Network pharmacology revealed that ZYS's anti‐aging effects are linked to the Focal Adhesion, Relaxin Signaling, PI3K‐Akt, TNF, and Apoptosis pathways. In vitro experiments further showed that ZYS modulates the expression of Elastin, Collagen I, Collagen III, Collagen IV, Collagen VII, and Decorin, underpinning its anti‐aging mechanisms. These findings suggest that ZYS is a promising adjunct for natural anti‐aging strategies.

## Author Contributions

**Yingpeng Tong:** writing – original draft, visualization, validation, software, methodology, formal analysis, data curation. **Xiangmei Ni:** visualization, validation, software, methodology, formal analysis, data curation. **Xinnan Jiang:** validation, software, methodology. **Ru Zhang:** methodology, formal analysis. **Pengcheng Guo:** methodology, formal analysis. **Chunbo Feng:** supervision, data curation, investigation, funding acquisition, conceptualization. **Jianxin Wang:** writing – review and editing, visualization, supervision, resources, project administration, investigation, funding acquisition, formal analysis.

## Funding

The authors have nothing to report.

## Ethics Statement

The protocol and consent form were approved on 20 December 2023 by the Ethics Committee of Shanghai Institute of Spice Research Co. Ltd. (2023‐LLSC‐GP231115~1121).

## Consent

We have stated that informed consent was obtained and provided an explanation in my methods section.

## Conflicts of Interest

The authors declare no conflicts of interest.

## Supporting information


**Figure S1:** Network of the main ingredients, pathways and targets of ZYS for anti‐skin aging. Nodes represent herbs, ingredients, targets and pathways were filled with Purple, blue, green, and orange, respectively.

**Table S1:** The results of network medicine analysis.

**Table S2:** The disease targets of skin aging.

**Table S3:** The results of molecular docking.

## Data Availability

The data that support the findings of this study are available from the corresponding author upon reasonable request.

## References

[1] S. H. Shin , Y. H. Lee , N. K. Rho , and K. Y. Park , “Skin Aging From Mechanisms to Interventions: Focusing on Dermal Aging,” Frontiers in Physiology 14 (2023): 1195272. 10.3389/fphys.2023.1195272 PMC10206231 37234413

[2] T. Chin , X. E. Lee , P. Y. Ng , Y. L. Lee , and O. Dreesen , “The Role of Cellular Senescence in Skin Aging and Age‐Related Skin Pathologies,” Frontiers in Physiology 14 (2023): 1297637. 10.3389/fphys.2023.1297637 PMC10703490 38074322

[3] F. Biskanaki , V. Kefala , A. C. Lazaris , and E. Rallis , “Aging and the Impact of Solar Ultraviolet Radiation on the Expression of Type I and Type VI Collagen,” Cosmetics 10, no. 2 (2023): 48.

[4] H. Knaggs and E. D. Lephart , “Enhancing Skin Anti‐Aging Through Healthy Lifestyle Factors,” Cosmetics 10, no. 5 (2023): 142.

[5] G. Bocheva , R. M. Slominski , and A. T. Slominski , “Environmental Air Pollutants Affecting Skin Functions With Systemic Implications,” International Journal of Molecular Sciences 24, no. 13 (2023): 10502. 10.3390/ijms241310502 PMC10341863 37445680

[6] T. Quan , “Molecular Insights of Human Skin Epidermal and Dermal Aging,” Journal of Dermatological Science 112, no. 2 (2023): 48–53. 10.1016/j.jdermsci.2023.08.006 PMC13155249 37661473

[7] Y. Liang , W. Su , and F. Wang , “Skin Ageing: A Progressive, Multi‐Factorial Condition Demanding an Integrated, Multilayer‐Targeted Remedy,” Clinical, Cosmetic and Investigational Dermatology 16 (2023): 1215–1229. 10.2147/CCID.S408765 PMC10182820 37192990

[8] S. J. Hwang , G. H. Ha , W. Y. Seo , C. K. Kim , K. Kim , and S. B. Lee , “Human Collagen Alpha‐2 Type I Stimulates Collagen Synthesis, Wound Healing, and Elastin Production in Normal Human Dermal Fibroblasts (HDFs),” BMB Reports 53, no. 10 (2020): 539–544. 10.5483/BMBRep.2020.53.10.120 PMC7607150 32843132

[9] S. W. Volk , Y. Wang , E. A. Mauldin , K. W. Liechty , and S. L. Adams , “Diminished Type III Collagen Promotes Myofibroblast Differentiation and Increases Scar Deposition in Cutaneous Wound Healing,” Cells, Tissues, Organs 194, no. 1 (2011): 25–37. 10.1159/000322399 PMC3128157 21252470

[10] X. Yuan , Q. Su , H. Wang , et al., “Genetic Variants of the COL4A3, COL4A4, and COL4A5 Genes Contribute to Thinned Glomerular Basement Membrane Lesions in Sporadic IgA Nephropathy Patients,” Journal of the American Society of Nephrology 34, no. 1 (2023): 132–144. 10.1681/ASN.2021111447 PMC10101589 36130833

[11] D. Rao , R. Maas , M. Cornelissen , J. Wetzels , and M. V. Geel , “Trigenic COL4A3/COL4A4/COL4A5 Pathogenic Variants in Alport Syndrome: A Case Report,” Nephron 148, no. 10 (2024): 724–730. 10.1159/000538587 PMC11407586 38547857

[12] H. Huang , T. Li , G. Ye , et al., “High Expression of COL10A1 Is Associated With Poor Prognosis in Colorectal Cancer,” Oncotargets and Therapy 11 (2018): 1571–1581. 10.2147/OTT.S160196 PMC5865565 29593423

[13] C. Ucaryilmaz Metin and G. Ozcan , “Comprehensive Bioinformatic Analysis Reveals a Cancer‐Associated Fibroblast Gene Signature as a Poor Prognostic Factor and Potential Therapeutic Target in Gastric Cancer,” BMC Cancer 22, no. 1 (2022): 692. 10.1186/s12885-022-09736-5 PMC9229147 35739492

[14] H. Wang , T. Ning , C. Song , et al., “Priming Integrin α5 Promotes Human Dental Pulp Stem Cells Odontogenic Differentiation due to Extracellular Matrix Deposition and Amplified Extracellular Matrix‐Receptor Activity,” Journal of Cellular Physiology 234, no. 8 (2019): 12897–12909. 10.1002/jcp.27954 PMC13483343 30556904

[15] J. Kim , M. B. Kim , J. G. Yun , and J. K. Hwang , “Protective Effects of Standardized Siegesbeckia Glabrescens Extract and Its Active Compound Kirenol Against UVB‐Induced Photoaging Through Inhibition of MAPK/NF‐κB Pathways,” Journal of Microbiology and Biotechnology 27, no. 2 (2017): 242–250. 10.4014/jmb.1610.10050 27880964

[16] Y. E. Choi , M. J. Song , M. Hara , et al., “Effects of Tenascin C on the Integrity of Extracellular Matrix and Skin Aging,” International Journal of Molecular Sciences 21, no. 22 (2020): 8693. 10.3390/ijms21228693 PMC7698786 33217999

[17] T. Wang , Y. Qin , J. Qiao , Y. Liu , L. Wang , and X. Zhang , “Overexpression of SIRT6 Regulates NRF2/HO‐1 and NF‐κB Signaling Pathways to Alleviate UVA‐Induced Photoaging in Skin Fibroblasts,” Journal of Photochemistry and Photobiology. B 249 (2023): 112801. 10.1016/j.jphotobiol.2023.112801 37897855

[18] J. Chen , Y. Li , Q. Zhu , et al., “Anti‐Skin‐Aging Effect of Epigallocatechin Gallate by Regulating Epidermal Growth Factor Receptor Pathway on Aging Mouse Model Induced by d‐Galactose,” Mechanisms of Ageing and Development 164 (2017): 1–7. 10.1016/j.mad.2017.03.007 28343910

[19] H. Liu , Q. Jian , K. Xue , et al., “The MEK/ERK Signalling Cascade Is Required for Sonic Hedgehog Signalling Pathway‐Mediated Enhancement of Proliferation and Inhibition of Apoptosis in Normal Keratinocytes,” Experimental Dermatology 23, no. 12 (2014): 896–901. 10.1111/exd.12556 25256290

[20] J. Wu , K. Wang , Q. Liu , et al., “An Integrative Pharmacology Model for Decoding the Underlying Therapeutic Mechanisms of Ermiao Powder for Rheumatoid Arthritis,” Frontiers in Pharmacology 13 (2022): 801350. 10.3389/fphar.2022.801350 PMC8905663 35281924

[21] H. Ke , X. Zhang , S. Liang , et al., “Study on the Anti‐Skin Aging Effect and Mechanism of Sijunzi Tang Based on Network Pharmacology and Experimental Validation,” Journal of Ethnopharmacology 333 (2024): 118421. 10.1016/j.jep.2024.118421 38880400

[22] D. L. Ndou , A. R. Ndhlala , N. T. Tavengwa , and N. E. Madala , “A Relook Into the Flavonoid Chemical Space of *Moringa oleifera* Lam. Leaves Through a Combination of LC‐MS and Molecular Networking,” Journal of Analytical Methods in Chemistry 2023, no. 1 (2023): 1327886. 10.1155/2023/1327886 PMC10545469 37790601

[23] C. B. Feng , C. Serre , W. M. Chen , I. Imbert , L. Zhu , and F. Ling , “Synergistic Effect of *Panax ginseng* , Polygonatum Cyrtonema, *Epiphyllum oxypetalum* , *Nelumbo nucifera* and *Osmanthus fragrans* Extracts on Skin Aging Regulation. From In Silico Predictions to In Vitro Outcome,” Heliyon 10, no. 5 (2024): e26131. 10.1016/j.heliyon.2024.e26131 PMC10915351 38449662

[24] J. Li , R. G. Wu , F. Y. Meng , et al., “Synergism and Rules From Combination of Baicalin, Jasminoidin and Desoxycholic Acid in Refined Qing Kai Ling for Treat Ischemic Stroke Mice Model,” PLoS One 7, no. 9 (2012): e45811. 10.1371/journal.pone.0045811 PMC3458908 23049867

[25] Z. Yunkun , Y. Rong , L. Lin , et al., “Medication Rule and Mechanism of Traditional Chinese Medicine in Treating Metabolism‐Associated Fatty Liver Disease Based on Bioinformatics Technology,” Digital Chinese Medicine 6, no. 3 (2023): 257–271.

[26] E. Axiotis , A. Angelis , L. Antoniadi , E. A. Petrakis , and L. A. Skaltsounis , “Phytochemical Analysis and Dermo‐Cosmetic Evaluation of Cymbidium sp. (Orchidaceae) Cultivation By‐Products,” Antioxidants 11, no. 1 (2021): 101. 10.3390/antiox11010101 PMC8772768 35052605

[27] A. L. Hopkins , “Network Pharmacology: The Next Paradigm in Drug Discovery,” Nature Chemical Biology 4, no. 11 (2008): 682–690. 10.1038/nchembio.118 18936753

[28] S. Li and B. Zhang , “Traditional Chinese Medicine Network Pharmacology: Theory, Methodology and Application,” Chinese Journal of Natural Medicines 11, no. 2 (2013): 110–120. 10.1016/S1875-5364(13)60037-0 23787177

[29] J. Wu and L. Pan , “Study on the Effect of *Pogostemon cablin* Benth on Skin Aging Based on Network Pharmacology,” Current Computer‐Aided Drug Design 18, no. 6 (2022): 459–468. 10.2174/1573409918666220901120750 36056869

[30] D. Morselli Gysi , Í. do Valle , M. Zitnik , et al., “Network Medicine Framework for Identifying Drug‐Repurposing Opportunities for COVID‐19,” Proceedings of the National Academy of Sciences of the United States of America 118, no. 19 (2021): e2025581118. 10.1073/pnas.2025581118 PMC8126852 33906951

[31] J. Ru , P. Li , J. Wang , et al., “TCMSP: A Database of Systems Pharmacology for Drug Discovery From Herbal Medicines,” Journal of Cheminformatics 6, no. 1 (2014): 13. 10.1186/1758-2946-6-13 PMC4001360 24735618

[32] UniProt Consortium , “UniProt: The Universal Protein Knowledgebase in 2025,” Nucleic Acids Research 53, no. D1 (2025): D609–D617. 10.1093/nar/gkae1010 PMC11701636 39552041

[33] K. Gallo , A. Goede , R. Preissner , and B.‐O. Gohlke , “SuperPred 3.0: Drug Classification and Target Prediction—A Machine Learning Approach,” Nucleic Acids Research 50, no. W1 (2022): W726–W731. 10.1093/nar/gkac297 PMC9252837 35524552

[34] E. Guney , J. Menche , M. Vidal , and A.‐L. Barábasi , “Network‐Based In Silico Drug Efficacy Screening,” Nature Communications 7, no. 1 (2016): 10331. 10.1038/ncomms10331 PMC4740350 26831545

[35] F. Cheng , I. A. Kovács , and A.‐L. Barabási , “Network‐Based Prediction of Drug Combinations,” Nature Communications 10, no. 1 (2019): 1197. 10.1038/s41467-019-09186-x PMC6416394 30867426

[36] J. Xia , X. He , W. Yang , et al., “Unveiling the Distribution of Chemical Constituents at Different Body Parts and Maturity Stages of *Ganoderma lingzhi* by Combining Metabolomics With Desorption Electrospray Ionization Mass Spectrometry Imaging (DESI),” Food Chemistry 436 (2024): 137737. 10.1016/j.foodchem.2023.137737 37857205

[37] X. Mo , L. Wang , C. Yu , and C. Kou , “Combined Metabolomics and Transcriptomics Analysis of the Distribution of Flavonoids in the Fibrous Root and Taproot of *Polygonatum kingianum* Coll. et Hemsl,” Genes 15, no. 7 (2024): 828. 10.3390/genes15070828 PMC11275391 39062607

[38] W. Liu , D.‐X. Yin , T. Zhang , Q. Qiao , Y.‐Q. Yang , and W.‐L. Wang , “Phytochemical Profiles and Antioxidant Activity of *Rehmannia glutinosa* From Different Production Locations,” Chemistry & Biodiversity 17, no. 8 (2020): e2000341. 10.1002/cbdv.202000341 32458564

[39] A. Daina , O. Michielin , and V. Zoete , “SwissTargetPrediction: Updated Data and New Features for Efficient Prediction of Protein Targets of Small Molecules,” Nucleic Acids Research 47, no. W1 (2019): W357–W364. 10.1093/nar/gkz382 PMC6602486 31106366

[40] Z. Wu , Y. Peng , Z. Yu , W. Li , G. Liu , and Y. Tang , “NetInfer: A Web Server for Prediction of Targets and Therapeutic and Adverse Effects via Network‐Based Inference Methods,” Journal of Chemical Information and Modeling 60, no. 8 (2020): 3687–3691. 10.1021/acs.jcim.0c00291 32687354

[41] M. Safran , I. Dalah , J. Alexander , et al., “GeneCards Version 3: The Human Gene Integrator,” Database 2010 (2010): baq020. 10.1093/database/baq020 PMC2938269 20689021

[42] S. Forli , R. Huey , M. E. Pique , M. F. Sanner , D. S. Goodsell , and A. J. Olson , “Computational Protein–Ligand Docking and Virtual Drug Screening With the AutoDock Suite,” Nature Protocols 11, no. 5 (2016): 905–919. 10.1038/nprot.2016.051 PMC4868550 27077332

[43] S. Yuan , H. C. S. Chan , and Z. Hu , “Using PyMOL as a Platform for Computational Drug Design,” WIREs Computational Molecular Science 7, no. 2 (2017): e1298.

[44] J. Jiménez , S. Doerr , G. Martínez‐Rosell , A. S. Rose , and G. De Fabritiis , “DeepSite: Protein‐Binding Site Predictor Using 3D‐Convolutional Neural Networks,” Bioinformatics 33, no. 19 (2017): 3036–3042. 10.1093/bioinformatics/btx350 28575181

[45] G. Dennis , B. T. Sherman , D. A. Hosack , et al., “DAVID: Database for Annotation, Visualization, and Integrated Discovery,” Genome Biology 4, no. 9 (2003): R60. PMC193660 12734009

[46] R. Saito , M. E. Smoot , K. Ono , et al., “A Travel Guide to Cytoscape Plugins,” Nature Methods 9, no. 11 (2012): 1069–1076. 10.1038/nmeth.2212 PMC3649846 23132118

[47] M. Pan , Y. Wu , C. Sun , H. Ma , X. Ye , and X. Li , “Polygonati Rhizoma: A Review on the Extraction, Purification, Structural Characterization, Biosynthesis of the Main Secondary Metabolites and Anti‐Aging Effects,” Journal of Ethnopharmacology 327 (2024): 118002. 10.1016/j.jep.2024.118002 38437890

[48] S. Song , F. Li , B. Zhao , M. Zhou , and X. Wang , “Ultraviolet Light Causes Skin Cell Senescence: From Mechanism to Prevention Principle,” Advanced Biology 9, no. 2 (2025): e2400090. 10.1002/adbi.202400090 39364703

[49] M. Kang , S. Park , S.‐R. Son , Y. Noh , D. S. Jang , and S. Lee , “Anti‐Aging and Anti‐Inflammatory Effects of Compounds From Fresh *Panax ginseng* Roots: A Study on TNF‐α/IFN‐γ‐Induced Skin Cell Damage,” Molecules 29, no. 22 (2024): 5479. 10.3390/molecules29225479 PMC11597146 39598869

[50] S. Srivastava , S. F. Huang , and M. S. Jagtap , “Assessment of the Effect of *Rehmannia glutinosa* Leaf Extract in Maintaining Skin Health: A Proof‐Of‐Concept, Double‐Blind, Randomized, Placebo‐Controlled Clinical Trial,” Clinical, Cosmetic and Investigational Dermatology 17 (2024): 863–875. 10.2147/CCID.S448928 PMC11034513 38651075

[51] Z.‐J. Guo , Y. Liu , J.‐Y. Yang , M.‐Y. Jin , P.‐W. Mao , and X.‐W. Zhou , “Evaluating the Application Potential of a Recombinant *Ganoderma* Protein as Bioactive Ingredients in Cosmetics,” Molecules 28, no. 7 (2023): 3272. 10.3390/molecules28073272 PMC10096787 37050035

[52] X. Zhang , Q. Chen , L. Chen , X. Chen , and Z. Ma , “Anti‐Aging in *Caenorhabditis elegans* of Polysaccharides From *Polygonatum cyrtonema* Hua,” Molecules 29, no. 6 (2024): 1276. 10.3390/molecules29061276 PMC10975160 38542911

[53] Q. Jiao , L. Zhi , B. You , G. Wang , N. Wu , and Y. Jia , “Skin Homeostasis: Mechanism and Influencing Factors,” Journal of Cosmetic Dermatology 23, no. 5 (2024): 1518–1526. 10.1111/jocd.16155 38409936

[54] J. Januszewski , A. Forma , J. Zembala , et al., “Nutritional Supplements for Skin Health—A Review of What Should be Chosen and Why,” Medicina 60, no. 1 (2024): 68. 10.3390/medicina60010068 PMC10820017 38256329

[55] Y. Li , Y. Ma , Y. Yao , et al., “Protective Effect of Isoquercitrin on UVB‐Induced Injury in HaCaT Cells and Mice Skin Through Anti‐Inflammatory, Antioxidant, and Regulation of MAPK and JAK2‐STAT3 Pathways,” Photochemistry and Photobiology 100, no. 5 (2024): 1507–1518. 10.1111/php.13919 38337181

[56] Y. Teng , Y. Fan , J. Ma , et al., “The PI3K/Akt Pathway: Emerging Roles in Skin Homeostasis and a Group of Non‐Malignant Skin Disorders,” Cells 10, no. 5 (2021): 1219. 10.3390/cells10051219 PMC8156939 34067630

[57] J. H. Chung , S. Kang , J. Varani , J. Lin , G. J. Fisher , and J. J. Voorhees , “Decreased Extracellular‐Signal‐Regulated Kinase and Increased Stress‐Activated MAP Kinase Activities in Aged Human Skin In Vivo,” Journal of Investigative Dermatology 115, no. 2 (2000): 177–182. 10.1046/j.1523-1747.2000.00009.x 10951233

[58] Y. Liu , L. Qu , S. Wan , Y. Li , and D. Fan , “Ginsenoside Rk1 Prevents UVB Irradiation‐Mediated Oxidative Stress, Inflammatory Response, and Collagen Degradation via the PI3K/AKT/NF‐κB Pathway In Vitro and In Vivo,” Journal of Agricultural and Food Chemistry 70, no. 50 (2022): 15804–15817. 10.1021/acs.jafc.2c06377 36472249

[59] J. H. Park , S. R. Kim , H. J. An , W. J. Kim , M. Choe , and J. A. Han , “Esculetin Promotes Type I Procollagen Expression in Human Dermal Fibroblasts Through MAPK and PI3K/Akt Pathways,” Molecular and Cellular Biochemistry 368, no. 1 (2012): 61–67. 10.1007/s11010-012-1342-7 22581442

[60] W. Wang , M. Zhong , R. Liu , et al., “Potential Cosmetic Applications of the Combined Extract of *Panax ginseng* , *Ganoderma lucidum*, *Cordyceps militaris*, and Several Asian Plants,” Journal of Cosmetic Dermatology 24, no. 8 (2025): e70343. 10.1111/jocd.70343 PMC12322945 40762221

