# Supplementary material for: Clinical Efficacy and Mechanisms of ZiyuShuang, an Anti‐Skin Aging Formula Identified Through Network Medicine Framework Analysis
Source: J Cosmet Dermatol. 2026 Sep 23;25(10):e71171. doi: 10.1111/jocd.71171 (PMC13599452; doi:10.1111/jocd.71171)
Supplement: Supplementary file 1 [file JOCD-25-e71171-s004.docx]

FIGURE S1. Network of the main ingredients, pathways and targets of ZYS for anti-skin aging. Nodes represent herbs, ingredients, targets and pathways were filled with Purple, blue, green, and orange, respectively.
